# Incorporating the effects of humidity in a mechanistic model of *Anopheles gambiae* mosquito population dynamics in the Sahel region of Africa

**DOI:** 10.1186/1756-3305-6-235

**Published:** 2013-08-09

**Authors:** Teresa K Yamana, Elfatih A B Eltahir

**Affiliations:** 1 Massachusetts Institute of Technology, Room 48–207, 15 Vassar Street, Cambridge, MA 02139, USA

**Keywords:** Malaria, Modelling, Humidity, Mosquito survival, Longevity, Desiccation, Anopheles

## Abstract

**Background:**

Low levels of relative humidity are known to decrease the lifespan of mosquitoes. However, most current models of malaria transmission do not account for the effects of relative humidity on mosquito survival. In the Sahel, where relative humidity drops to levels <20% for several months of the year, we expect relative humidity to play a significant role in shaping the seasonal profile of mosquito populations. Here, we present a new formulation for *Anopheles gambiae sensu lato* (*s*.*l*.) mosquito survival as a function of temperature and relative humidity and investigate the effect of humidity on simulated mosquito populations.

**Methods:**

Using existing observations on relationships between temperature, relative humidity and mosquito longevity, we developed a new equation for mosquito survival as a function of temperature and relative humidity. We collected simultaneous field observations on temperature, wind, relative humidity, and anopheline mosquito populations for two villages from the Sahel region of Africa, which are presented in this paper. We apply this equation to the environmental data and conduct numerical simulations of mosquito populations using the Hydrology, Entomology and Malaria Transmission Simulator (HYDREMATS).

**Results:**

Relative humidity drops to levels that are uncomfortable for mosquitoes at the end of the rainy season. In one village, Banizoumbou, water pools dried up and interrupted mosquito breeding shortly after the end of the rainy season. In this case, relative humidity had little effect on the mosquito population. However, in the other village, Zindarou, the relatively shallow water table led to water pools that persisted several months beyond the end of the rainy season. In this case, the decrease in mosquito survival due to relative humidity improved the model’s ability to reproduce the seasonal pattern of observed mosquito abundance.

**Conclusions:**

We proposed a new equation to describe *Anopheles gambiae s*.*l*. mosquito survival as a function of temperature and relative humidity. We demonstrated that relative humidity can play a significant role in mosquito population and malaria transmission dynamics. Future modeling work should account for these effects of relative humidity.

## Background

**Figure 1:**
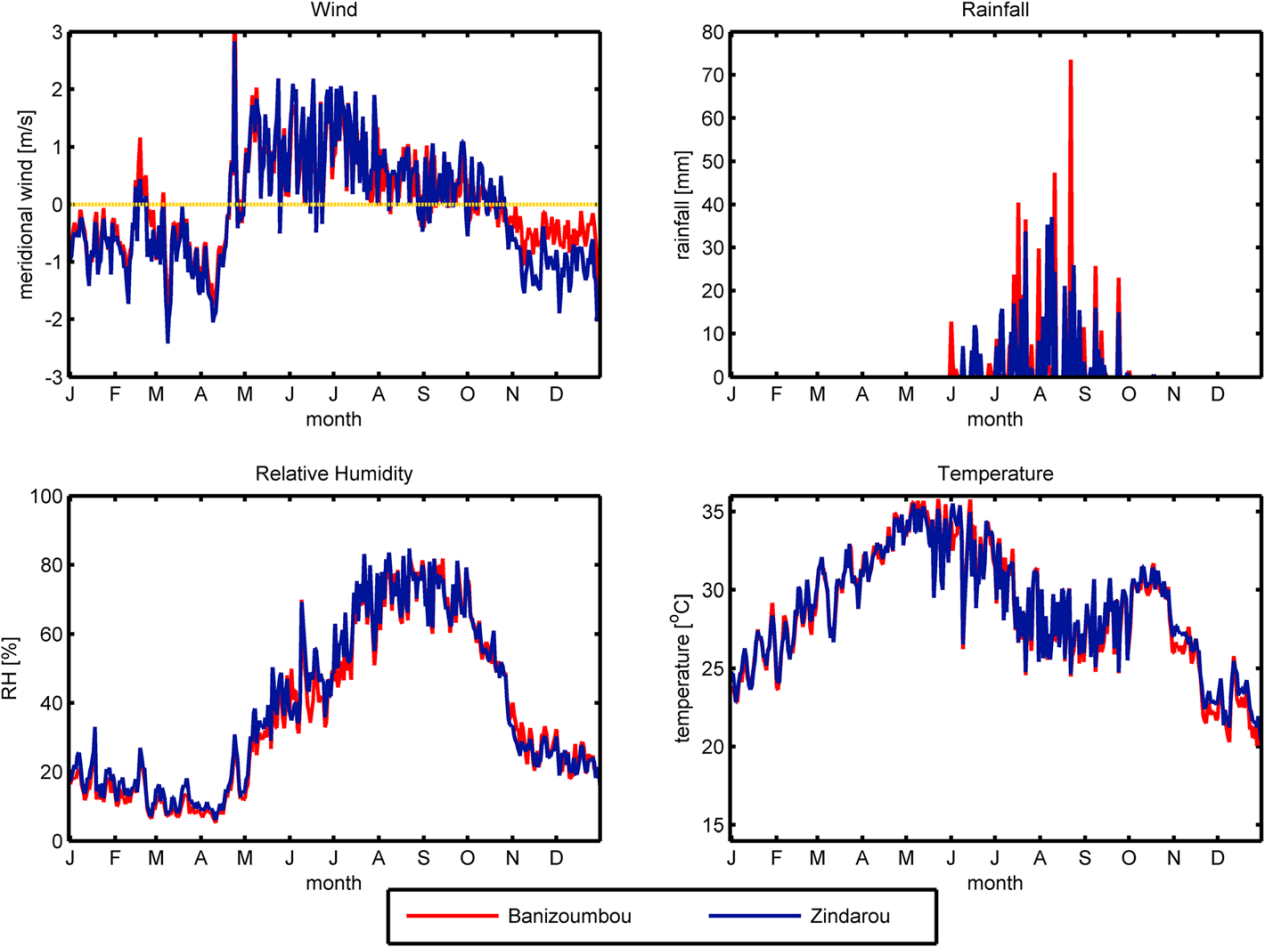
**Climate in Banizoumbou and Zindarou.** Ground observations of daily average meridional wind speed, daily total rainfall, and daily averages of relative humidity and temperature are shown for Banizoumbou (red) and Zindarou (blue) for the year 2006. Postive values (above the yellow line) indicate wind blowing from the south while negative values (below the yellow line) indicate wind from the north.

In this paper, we investigated the effects of relative humidity on simulated mosquito population dynamics in two villages in Niger, West Africa. These two villages, Banizoumbou (13.53° N, 2.66° E) and Zindarou (13.43° N, 2.92° E), were the subject of extensive field activities and numerical model simulations using HYDREMATS (Hydrology, Entomology and Malaria Transmission Simulator), a spatially explicit, mechanistic model of malaria transmission [1,2]. The two villages are approximately 30 km apart, and there is little difference in climate between the two. Climate data for 2006 are shown in Figure 1. The rainy season in the region is dictated by the migration of the West African Monsoon. From approximately November through April, Harmattan winds from the Northeast bring dry air from the Sahara desert. From May through October, the monsoon brings moist air from the Southwest [3]. This shift in wind direction as measured in Banizoumbou and Zindarou in 2006 is shown in the upper left panel of Figure 1, where positive values of the meridional wind speed indicate wind blowing from the south, while negative values indicate wind blowing from the north. The moist air from the south leads to high relative humidity and rainfall as shown in the lower left and upper right panels of Figure 1, respectively.

**Figure 2:**
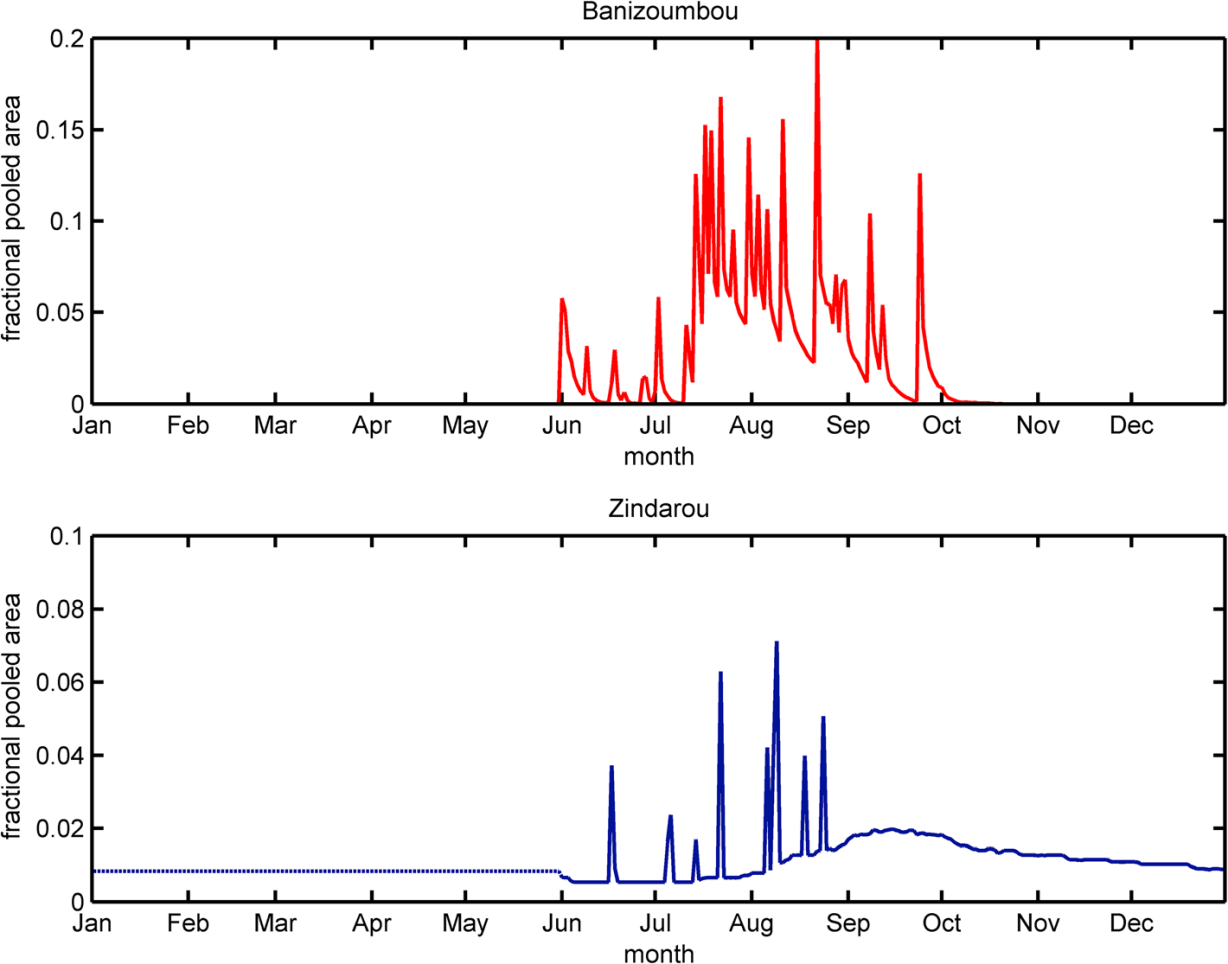
**Simulated water pools as a fraction of total surface area in Banizoumbou (top) and Zindarou (bottom) in 2006.** The dotted line from Jan – May in Zindarou indicates the assumption that permanent pools simulated in December persist through the dry season.

Despite similarities in climate, the two villages differ in their water availability. Banizoumbou is a typical Sahelian village with a deep water table (~25 meters), and surface water pools quickly dry up at the end of the rainy season. In contrast, Zindarou is located in the floodplain of an abandoned river system and is thus at a lower elevation (water table depth ~2.5 meters), allowing the groundwater to penetrate the land surface. This surface expression of ground water produces a wetter environment than is typically found in the Sahel allowing for high levels of mosquito breeding. The water pools in Zindarou persist for several months beyond the end of the rainy season, potentially extending the mosquito breeding season. Hydrological simulations of the two villages reflected this difference in pool availability, shown in Figure 2[1].

**Figure 3:**
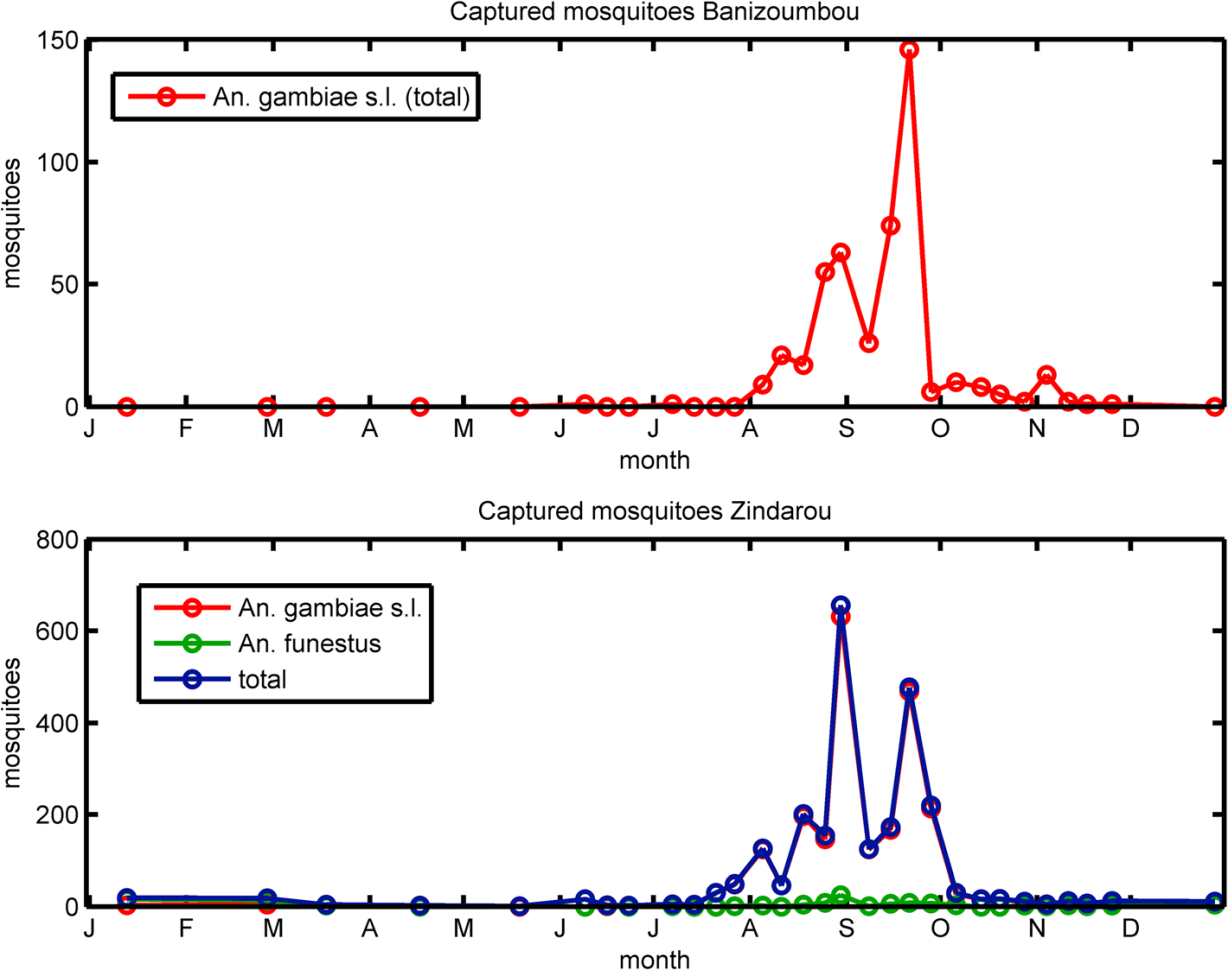
Mosquitoes captured by CDC light traps in Banizoumbou (top) and Zindarou (bottom) in 2006.

Mosquito collections were conducted as described in Bomblies *et al*. [2] and Bomblies *et al*. [1]. Six CDC light traps were deployed overnight in each village once a month from December – May (dry season), and weekly from June – November (wet season). Female *Anopheles gambiae s*.*l*. and *Anopheles funestus* specimens, the important malaria vectors in this region, were identified by microscopy and counted. While the mosquito captures began in 2005, we focus here on 2006, which is the year for which complete climate information was available. As shown in Figure 3, the observed number of anophelines followed a distinct seasonal cycle, increasing after the onset of monsoon rains in June, peaking in September, and returning to low levels by late October. No *An*. *funestus* were found in Banizoumbou, but a small number were found in Zindarou. The anopheline population in Zindarou did not persist beyond the end of the rainy season despite the continued availability of water pools for breeding. Bomblies *et al*. [1] hypothesized that the observed drop in mosquito population, despite continued availability of breeding sites was due to the lack of nutrient availability for larvae, as the decline in population coincided with the end of millet pollination. Here, we investigate whether the seasonal drop in humidity at the end of the rainy season could play a role in limiting mosquito populations.

### Effects of humidity on mosquito longevity

Mosquitoes, like all insects, have a limited range of tolerable temperature and humidity [4]. The high surface area to volume ratio of mosquitoes makes them especially sensitive to desiccation at low humidity levels.

Gaaboub *et al*. [5] compared the survival of groups of female *Anopheles pharoensis* mosquitoes at 20°, 26° and 30°C and found little difference in longevity between 50% and 90% relative humidity (RH) conditions at a given temperature. Bayoh and Lindsay [6] measured the longevity of *An*. *gambiae sensu stricto* (*s*.*s*.) at 40%, 60%, 80% and 100% RH at 5°C intervals from 5°C to 40°C. Under the assumption that daily probability of survival is independent of mosquito age, there was little difference in survival between 60-100% RH, but survival was slightly reduced at 40% RH.

Molecular biology techniques applied to *An*. *gambiae s*.*s*. held at 42% RH [7] and 30% RH [8] found the mosquitoes had undergone physiologic responses to desiccation stress, decreasing their water loss. Mosquitoes held without food or water survived for an average of 15.6 hours at 30% RH compared to 26.2 hours at 70% RH [8].

Recent studies on mosquito desiccation showed that extremely low levels of RH are fatal to mosquitoes when maintained for periods on the order of hours. These studies placed mosquitoes in vials without access to food or water and added a desiccant to reduce RH levels that are generally kept at <10% RH but not exactly specified. Several such studies found that no *An*. *gambiae s*.*s*. or *An*. *arabiensis* females survived for an entire day at <10% RH [9,10] or <20% RH [7]. In a similar study, a small number of mosquitoes survived up to 30 hours at <10% RH, and acclimation to hot and dry conditions was shown to increase desiccation resistance [11]. However, in another study using the offspring of field captured mosquitoes held at <10% RH, 15% of S form *An*. *gambiae s*.*s*. and 23% of M form *An*. *gambiae s*.*s*. females survived for over 1 day, with 2 out of 30 M form individuals surviving for over 2 days [12], suggesting that wild mosquitoes in arid regions may have higher desiccation resistance than laboratory colonies.

In summary, *An*. *gambiae* longevity does not appear to be substantially affected by relative humidity at ranges greater than 60%, but RH <10% is fatal, usually within hours. There is very little information on mosquito longevity in the range 10-40% RH.

### Mosquito survival in malaria models

Many mechanistic models of malaria transmission, including HYDREMATS, use the Martens equation for survival as a function of temperature [13-16]:

(1) $$p\left(T\right)=\exp\left(\frac{-1}{-4.4+1.31T-0.03T^{2}}\right)$$

where T is the daily average air temperature in degrees Celsius. This function gives maximum longevity in the range of 20-25°C, and severe mortality at temperatures below 10°C and above 35°C. This curve was formed based on three data points [17].

The experiments relating survival to temperature conducted by Bayoh [6] led to the development of two new formulations of survival probability of *Anopheles gambiae* to temperature, one by Ermert *et al*. [16] and another by Mordecai *et al*. [18], shown in Additional file 1. The accuracy of these two formulations and the Martens equation were recently evaluated by Lunde *et al*. [19].

Relative humidity has recently been incorporated into several models. Parham *et al*. [20] developed a survival curve based on Bayoh’s survival data [6]. Ermert *et al*. [16] account for humidity in the Liverpool Malaria Model by subtracting 10% from the daily probability of survival when 10 day accumulated rainfall is below 10 mm. Lunde *et al*. [21] use Bayoh’s survival data by fitting a survival curve for each measured value of RH (40%, 60%, 80% and 100%), further adjusted by mosquito size and age. While these formulations for mosquito survival rates are improvements on previous formulations that considered only temperature, they do not reliably capture the effect of very low values of relative humidity (<40% RH) such as those observed during the dry season in the Sahel on mosquito survival. Here, we propose a new equation for mosquito survival incorporating current knowledge on the effects of relative humidity and temperature on survival.

## Methods

### Development of new survival equation

We based our formulation of anopheline survival on an existing relationship between temperature and survival, *p*(*T*). In this paper, we use the Martens equation [17] described above for *p*(*T*). However, this equation can be substituted by an alternative formulation such as those evaluated by Lunde *et al*. [19], as discussed in Additional file 1. We make the assumption that the survival equation, *p*(*T*), accurately describes *Anopheles gambiae* survival at high and moderate levels of relative humidity.

We then add the effect of relative humidity by multiplying the survival by a relative humidity stress factor *S*. The humidity stress factor was developed using the observations reviewed in the previous section, and listed in Table 1. As we summarized in the previous section, there are some data reported for mosquito survival at RH values ≥40% and < 10%. We assume that mosquitoes feel no humidity stress at daily average relative humidity greater than or equal to some value *RH*_*S*_ (*S* = 0), and are stressed to the point of being unable to survive an entire day at critical daily average relative humidity *RH*_*C*_ (*S* = 1). In the absence of data measuring mosquito longevity at relative humidity between 10% and 40%, we assume S decreases linearly from 1 at *RH*_*C*_ to 0 at *RH*_*S*_.

**Table 1:** Observation of mosquito longevity used for development of relative humidity stress factor

**Species**	**Temperature**	**RH**	**Reference**
*An*. *pharoensis*	20°C-30°C	50%, 90%	Gaaboub *et al*., [5]
*An*. *gambiae s*.*s*.	5°C−40°C	40-100%	Bayoh, [6]
*An*. *gambiae s*.*s*.	28°C	<20%, 42%	Liu et al., [7]
*An*. *gambiae s*.*s*.	27°C	30%, 70%	Wang *et al*., [8]
*An*. *gambiae s*.*s*.	28°C	<10%	Gray and Bradley, [9]
*An*. *arabiensis*	28°C	<10%	Gray and Bradley, [9]
*An*. *gambiae s*.*s*.	27°C	<10%	Gray *et al*., [11]
*An*. *gambiae s*.*s*.	26°C	5%	Fouet *et al*., [10]
*An*. *gambiae s*.*s*.	28°C	<10%	Lee *et al*., [12]

The stress factor is then defined as follows:

(2) $$S=\left\{\begin{array}{c} 1,\mathrm{RH}<RH_{C}\\ \;\frac{RH_{S}-\mathrm{RH}}{RH_{S}-RH_{C}},RH_{C}\leq \mathrm{RH}\leq RH_{S}\\ \;0,\mathrm{RH}>RH_{S} \end{array}\right)\;$$

The new equation for *An*. *gambiae* survival is given by:

(3) $$p\left(T,\mathrm{RH}\right)=p\left(T\right)\times \left(1-S\right)$$

This assumes that temperature and relative humidity act independently on mosquito survival.

**Figure 4:**
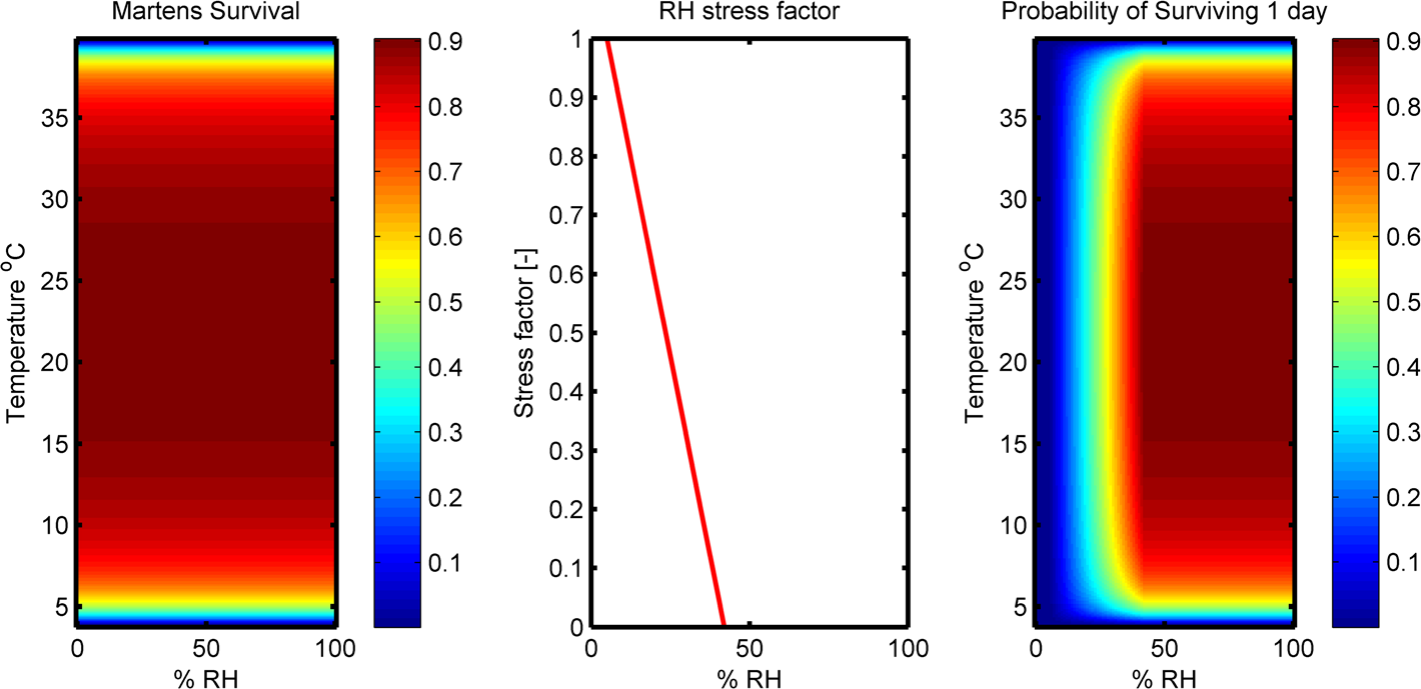
Martens survival equation (left), RH stress index (center) and daily survival probability of mosquitoes using the newly developed formula (right).

Mosquito survival as a function of temperature and RH are shown for *RH*_*S*_ = 42% and *RH*_*C*_ = 5% is shown in Figure 4. *RH*_*S*_ was set at 42% RH in order to reflect a decrease in longevity observed by Bayoh [6] at 40% RH compared to values ≥ 60% RH, and evidence that mosquitoes at 42% RH showed physiological signs of stress [7]. *RH*_*C*_ was set at 5% as several of the desiccation studies found that RH < 10% killed all mosquitoes in one day [9,10]. While most of the experiments conducted to date relating mosquito survival to relative humidity and temperature focused on *An*. *gambiae s*.*s*., we make the assumption that our model is valid for the *An*. *gambiae s*.*l*. complex. However, parameter values can be adjusted to reflect regional or species-specific differences in tolerance to arid conditions, or based on improved knowledge on mosquito longevity at RH <40%.

The first panel of Figure 4 shows the Martens survival curve (Equation 1), which is a function of temperature only. The second panel shows the RH stress factor calculated above (Equation 2). The third panel of Figure 4 shows the new equation for mosquito survival as a function of temperature and relative humidity (Equation 3).

**Figure 5:**
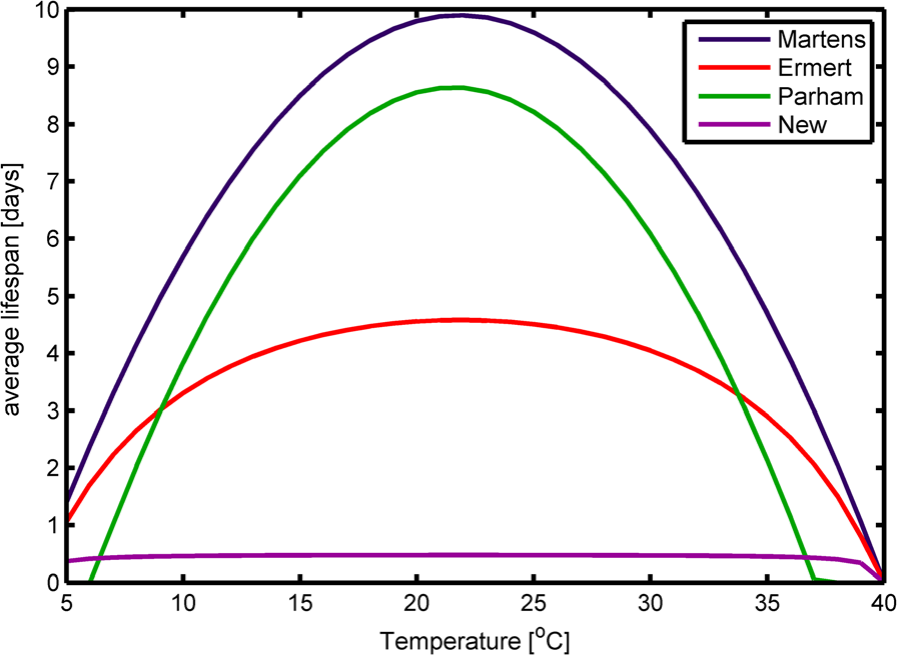
Average lifespan at 10% RH using various equations for mosquito survival.

The average lifespan of a mosquito can be calculated from the daily probability of survival: Lifespan = 1/-ln(p). Figure 5 shows a comparison of average lifespan using the Martens, Ermert Liverpool Malaria Model (dry season) and Parham equations and the new equation developed here when RH is held constant at 10%. At moderate temperatures (15-30°C), only the new equation reflects the lethal effects of extremely low RH observed in desiccation studies [9-12].

We do not explicitly consider the possibility of aestivation, by which mosquitoes survive for long periods during the dry season. This mechanism for survival has been observed in several instances in *An*. *gambiae* in the Sahel [22,23], but is still not well understood.

### Testing new survival equation

We tested the impact of the new survival equation in HYDREMATS, a mechanistic model of malaria transmission developed to simulate village-scale responses of malaria transmission to interannual climate variability in semi-arid desert fringe environments such as the Sahel, which has been used in a number of recent modeling studies in this region [1,24-27]. The development of HYDREMATS is described in detail in Bomblies *et al*. [2], and key features of the model are included in Additional file 2. The model provides explicit representation of the spatial determinants of malaria transmission. HYDREMATS can be separated into two components: the hydrology component which explicitly represents pooled water available to *Anopheles* mosquitoes as breeding sites, and the entomology component, which is an agent-based model of disease transmission.

In the hydrology component, rainfall is partitioned between runoff and infiltration, with soil and vegetation properties strongly influencing the partition between these two processes. Uptake of soil water from evapotranspiration is calculated based on climatic variables. Overland flow is modelled using a finite difference solution, and flow velocity is calculated as a function of friction slope, flow depth, and a distributed roughness parameter derived from soil characteristics and vegetation type. The overland flow process is of critical importance for the modelling of water pool formation. The regional unconfined aquifer is represented using a lumped model in which groundwater table fluctuations are simulated. The depth to the water table varies from cell to cell and is a function of topography. The hydrology component of HYDREMATS simulates the spatial distribution of water depths and temperatures for each grid cell, for each timestep. These distributions serve as the inputs for the entomology component of the model [2]. The hydrology component of the model was validated in Banizoumbou and Zindarou, Niger by comparing simulation results to measured soil moisture values ([2], Figure nine; [1], Figure four), groundwater level ([1], Figure five), and observed water pools ([2], Figures ten and eleven).

The entomology component of HYDREMATS simulates individual mosquito and human agents. Human agents are immobile, and are assigned to village residences, as malaria transmission in this region occurs primarily at night when humans are indoors [28]. Mosquito agents have a probabilistic response to their environment based on a prescribed set of rules governing dispersal and discrete events including development of larval stages, feeding, egg-laying and death [2]. Simulated mosquito numbers compared well with field captures of mosquitoes using CDC light traps ([2], Figures fourteen and fifteen; [1], Figure eight).

Simultaneous field observations on temperature, wind, relative humidity, and rainfall were collected for Banizoumbou and Zindarou. In order to assess the effect of relative humidity on mosquito population dynamics, we conducted simulations using observed environmental data for the year 2006. For each village, we conducted one simulation using the original Martens equation for mosquito longevity as a function of temperature only, and 3 simulations using the new equation incorporating temperature and relative humidity using RH_S_ = 42%, 40% and 35%, all at *RH*_*C*_ = 5%. We also conducted a simulation for each village with RH_S_ = 42% and *RH*_*C*_ = 0%.

## Results

**Figure 6:**
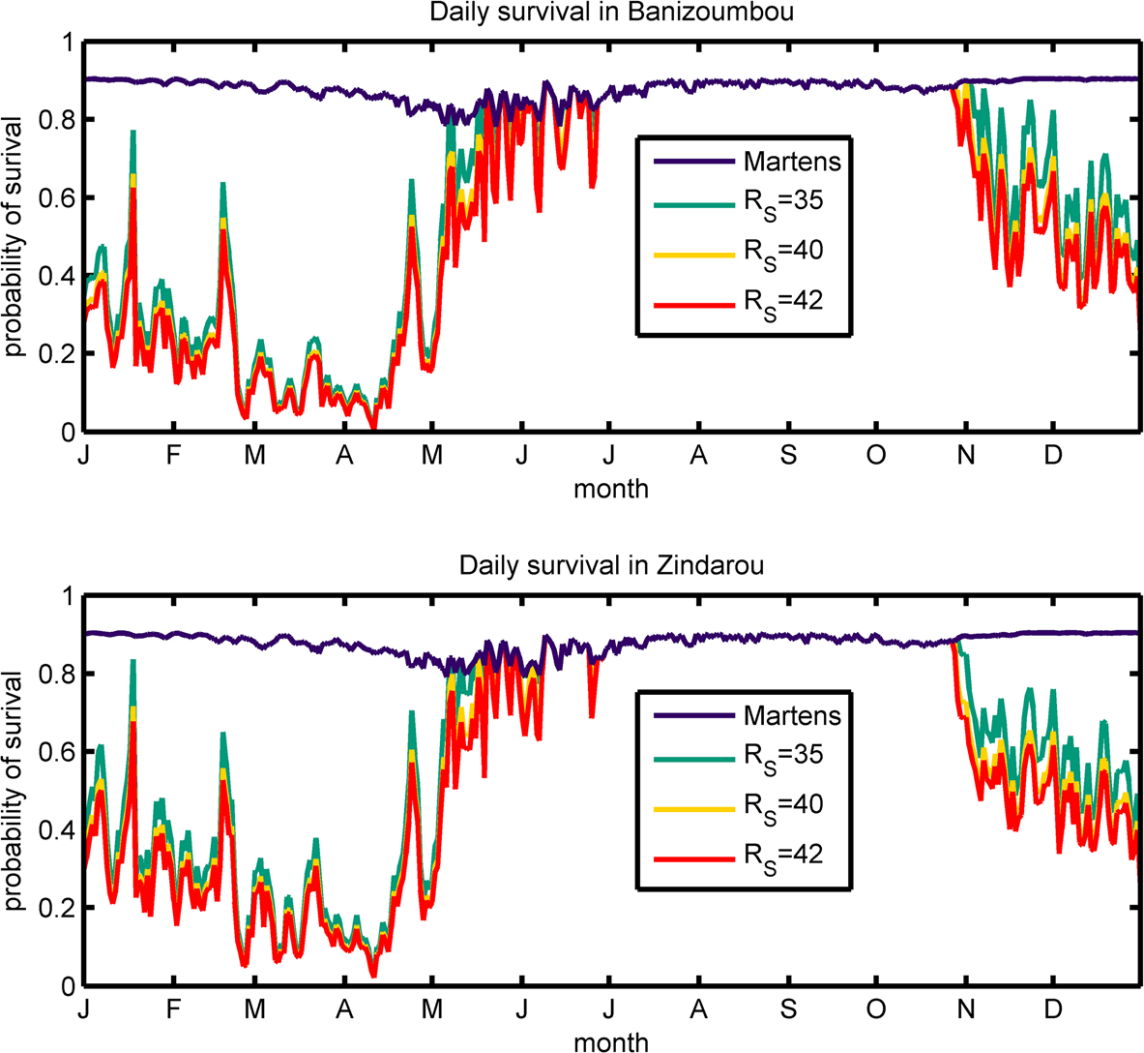
Daily probability of survival of mosquitoes using temperature and relative humidity data from Banizoumbou and Zindarou.

The daily probability of mosquito survival calculated using the Martens equation and the new equation incorporating relative humidity are shown for each village in Figure 6. When mosquito survival depended only on temperature, as shown in the blue line of Figure 6, there was little seasonal variation in the probability of survival, which ranged between 0.78 and 0.90, reaching a minimum between April and July. This figure shows that temperature cannot explain the observed decrease in mosquito population at the end of the wet season. When relative humidity was included in the calculation of mosquito survival, we observed a highly seasonal pattern. During most of the wet season, relative humidity is high and therefore does not contribute to mosquito mortality. However, during the dry season, RH significantly reduces mosquito survival to values as low as 0.03 in late-March and early-April where relative humidity falls below 10%. The value of RH_S_ determines the extent of RH related mortality.

**Figure 7:**
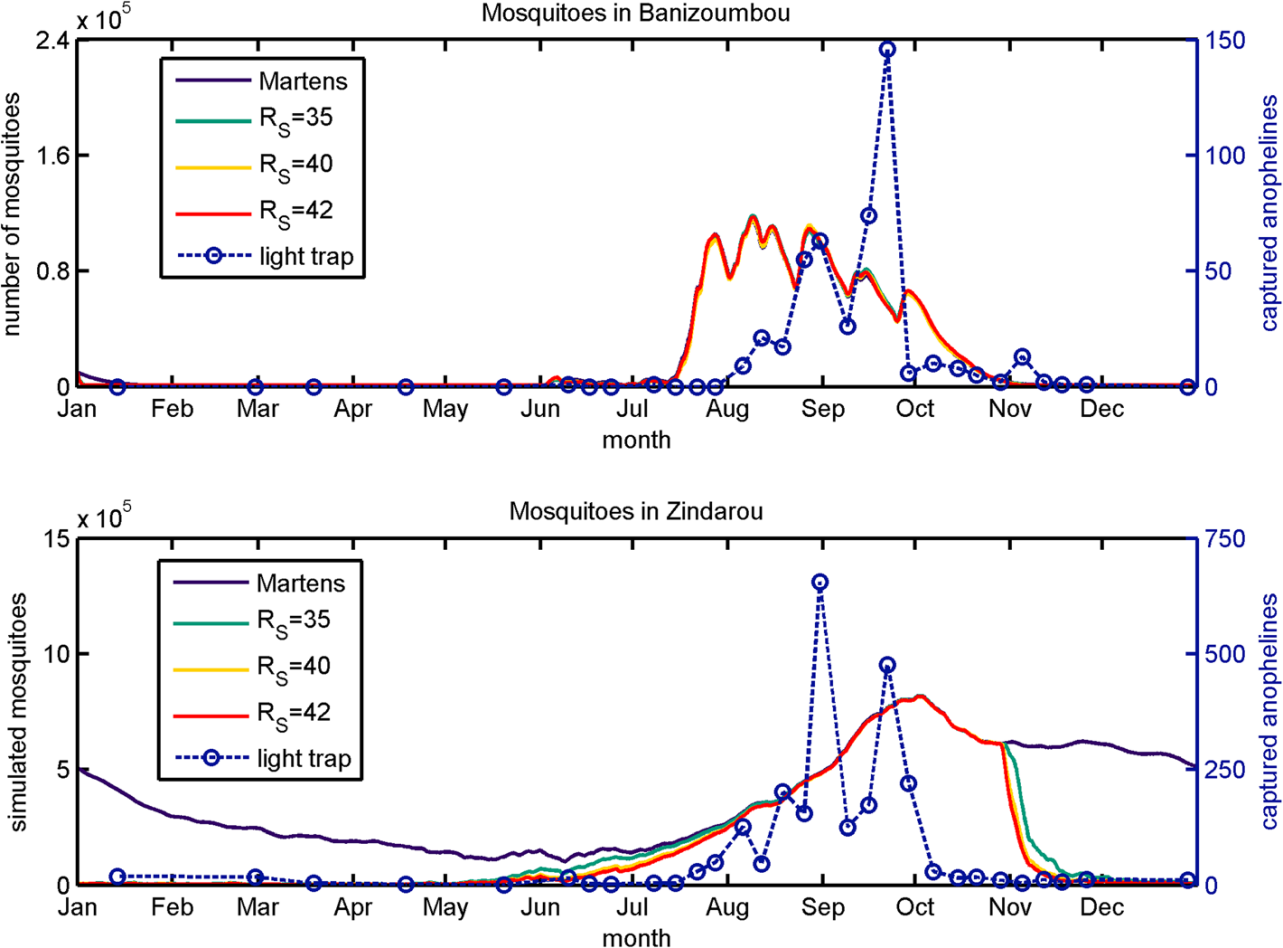
**Simulated mosquitoes in Banizoumbou (top) and Zindarou (bottom) using the differing values of RH**_**S**_**.** Mosquitoes captured by light traps are shown by the dashed line.

The size of the mosquito population in each simulation is shown in Figure 7. In Banizoumbou (Figure 7, top panel), mosquito population levels were closely tied to rainfall. There was little persistence of water pools beyond the end of the rainy season. Since the decrease in rainfall precedes the decrease in humidity, the addition of a stress factor at low levels of RH had minimal effect on mosquito populations. By contrast in Zindarou, where water pools persist for several months after the end of the rainy season, the size of the mosquito population was not limited by water availability. Temperature was also not a limiting factor at the end of the rainy season; in the simulation using the Martens equation for mosquito survival as a function of temperature, the mosquito population remained at high levels for the duration of the simulation (Figure 7, bottom panel, blue line). However, the incorporation of relative humidity into mosquito survival dramatically reduced the number of mosquitoes, beginning in late-October when the RH plummets. The choice of *RH*_*S*_ affected the results. In the simulations using *RH*_*S*_ = 40% and 42%, the mosquito populations dropped dramatically starting on October 29^th^. When *RH*_*S*_ was set to 35%, there was no change in mosquito populations until November 4^th^, and the drop was somewhat more gradual than in the in the simulations with higher *RH*_*S*_. There was little difference between simulations with *RH*_*C*_ = 5% and *RH*_*C*_ = 0% (see Additional file 3).

The incorporation of relative humidity into simulations of mosquito populations substantially decreases mosquito longevity. In cases such as Zindarou where breeding sites are available beyond the end of the wet season, the drop in relative humidity could explain, at least in part, the rapid decline of the mosquito population in field observations. However, the timing of the decline of mosquitoes in the simulation (late October) occurred approximately four weeks after the decrease in captured mosquitoes (late September/early October), indicating that other factors likely played a role in limiting mosquito numbers.

## Discussion

The adverse effects of low humidity on mosquito longevity have been known for decades [29]. Here, we have taken a commonly used equation for mosquito survival as a function of temperature and added the effects of relative humidity. While other researchers have incorporated humidity into their models [16,20,21], our equation is unique in that it reflects the fatal effect of the extremely low values of relative humidity that are observed during the dry season in the Sahel. Using evidence from mosquito survival studies, we assumed that relative humidity does not affect survival rates at high and moderate values of RH, but at a value *RH*_*S*_ (~42% RH), survival decreases until a critical value *RH*_*C*_ (~5% RH) where it is assumed that no individual can survive for longer than 24 hours. In the two villages of the Sahel described here, daily averages of relative humidity remained below 30% for the majority of the dry season.

The primary mode of variability in mosquito populations in these villages features two distinct seasons; a wet season with a high population of mosquitoes and relatively high malaria transmission (July-November) and a dry season with a low population of mosquitoes and low malaria transmission (December – June). When we simulate mosquito populations using HYDREMATS parameterized with the Martens survival equation, it reproduces this mode of variability in Banizoumbou, where mosquito breeding sites were not available beyond the wet season, but it fails to reproduce the same mode in Zindarou, where breeding sites persist into the dry season. However, when we incorporate the constraints on survival due to humidity developed here into HYDREMATS, the model reproduces this observed mode of variability in both villages.

While the equation for mosquito survival developed here improved the model’s ability to simulate the observed seasonal pattern of mosquitoes in Zindarou, the timing of the decline of captured mosquitoes preceded the drop in relative humidity by approximately 4 weeks, indicating that other factors must be playing a role in the mosquito decline. Other potential factors involved in this decline in mosquitoes could include a lack of nutrient availability for larvae and establishment of predator populations in the long-lasting water pools, which can be represented in HYDREMATS but were not included in the simulations for this study. Bomblies *et al*. [1] noted that the decline in mosquito population corresponded with the harvest of millet crops and hypothesized that aquatic stage mosquitoes may have depended on the availability of millet pollen. While anopheline larvae were found in the persistent water pools, it is possible that these pools become less attractive as breeding sites as the rainy season progresses, perhaps due to increased vegetation, turbidity or predator activity. Another possible explanation for the decline in mosquito captures could be the triggering of aestivation, where mosquitoes retreat to sheltered locations and cease regular activities, leading to a decrease in captured mosquitoes despite the continued presence of water pools.

In addition to the dramatic reduction in the mosquito population simulated in Zindarou as a result of low RH, mosquito longevity in individual mosquitoes plays an important role in malaria transmission dynamics. In order to transmit the parasite, a mosquito must survive long enough to bite an infected person, surpass the extrinsic incubation period of the parasite, roughly 6–10 days in warm climates [30], and then bite a second (uninfected) person. This amplifies the effect of shortened lifespan, such that even a small decrease in lifespan can have a very significant effect on malaria transmission [31].

Humidity may also play a role in determining future environmental suitability for malaria transmission under climate change. Climate change impact studies often focus on rainfall and temperature (eg. [32,33]), but relative humidity may change as well. Observations over the last few decades indicate that while global mean specific humidity is increasing, the accompanying increase in temperatures means there has been little change in relative humidity [34,35]. However, statistically significant changes in relative humidity have been observed on regional scales between 1975 and 2005 [36]. Current general circulation models indicate that increasing levels of greenhouse gas emissions could lead to substantial disruption of the West African Monsoon [37], which could alter spatial and temporal patterns of relative humidity in the Sahel.

## Conclusion

We proposed a new equation to describe mosquito survival as a function of temperature and relative humidity. We demonstrated that relative humidity can play a significant role in mosquito survival and malaria transmission dynamics. In the Sahel, where dry season RH regularly drops to levels known to significantly decrease mosquito longevity, relative humidity can be as important as temperature and rainfall in determining the environmental suitability for mosquitoes and malaria transmission. The primary mode of variability in mosquito populations in these villages features two distinct seasons; a wet season with a high population of mosquitoes and relatively high malaria transmission and a dry season with a low population of mosquitoes and low malaria transmission. We showed that when we simulate mosquito populations using HYDREMATS parameterized with the Martens survival equation, it fails to reproduce the same mode in Zindarou, where breeding sites persist into the dry season. However, when we incorporate the constraints on survival due to humidity developed here into HYDREMATS, the model reproduces this observed mode of variability in both villages. Future modeling work should therefore account for these effects of relative humidity.

## Competing interests

The authors declare they have no competing interests.

## Authors’ contributions

TKY conducted the study and drafted the manuscript. EABE supervised the study, provided advice for study design and implementation, and participated in writing the final version of the manuscript. All authors read and approved the final manuscript.

## Supplementary Material

Additional file 1 Alternative formulations for daily probability of survival as a function of temperature. Click here for file

Additional file 2 Additional information on HYDREMATS model. Click here for file

Additional file 3 **Simulated mosquitoes in Banizoumbou and Zindarou with *RH***_***C***_**= 5% and *RH***_***C***_** = 0%.** Click here for file
