# Supplementary material for: Incorporating the effects of humidity in a mechanistic model of Anopheles gambiae mosquito population dynamics in the Sahel region of Africa
Source: Parasit Vectors. 2013 Aug 9;6:235. doi: 10.1186/1756-3305-6-235 (PMC3750695; doi:10.1186/1756-3305-6-235)
Supplement: Additional file 1 — Alternative formulations for daily probability of survival as a function of temperature. [file 1756-3305-6-235-S1.pdf]

Additional File 1: Alternative formulations for daily probability of survival as a function of temperature

In this paper, we used the Martens equation [1] for daily probability of survival as a function of temperature, *p(T)*. However the equation we developed can substitute any other formulation for *p(T)*. Here, we consider two other equations that have recently been developed using new survival data [2], which we will refer to as Bayoh-Ermert [3] and Bayoh-Mordecai [4]. The three survival curves are shown in Figure S1.1.

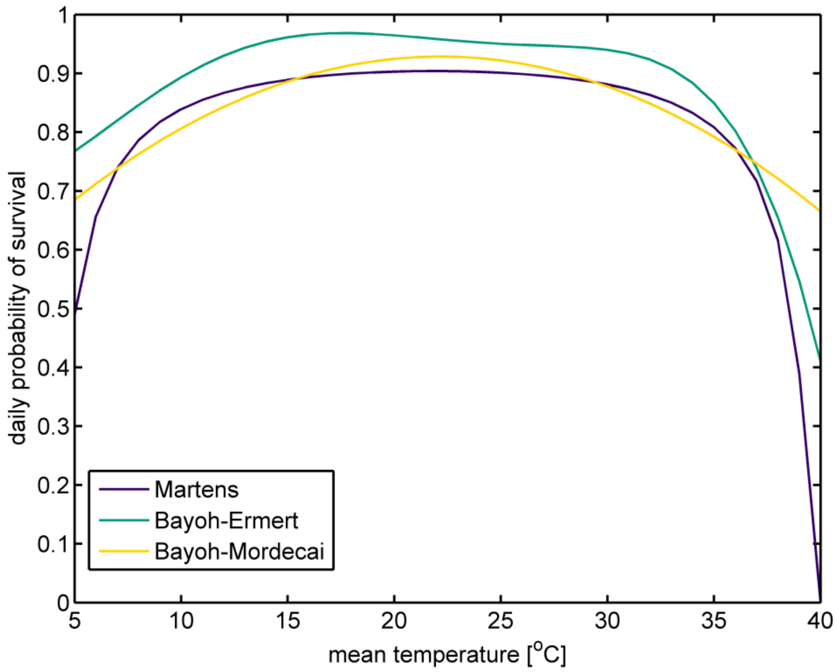

Figure S1.1: Daily probability of survival, p(T).

Figure S1.2 shows the daily probability of survival as a function of temperature and relative humidity, *p(T,RH)*, using the three different equations for *p(T)* and adjusted for relative humidity using the method presented in this paper using parameters RH_S=42% and RH_C=5%. In the temperature range observed in Banizoumbou and Zindarou (20-35°C), the three *p(T)* curves give similar survival probabilities, so there is little difference in the calculated *p(T,RH)*. The Bayoh-Ermert equation leads to higher survival during the wet season, but the effects of relative humidity remain largely unchanged.

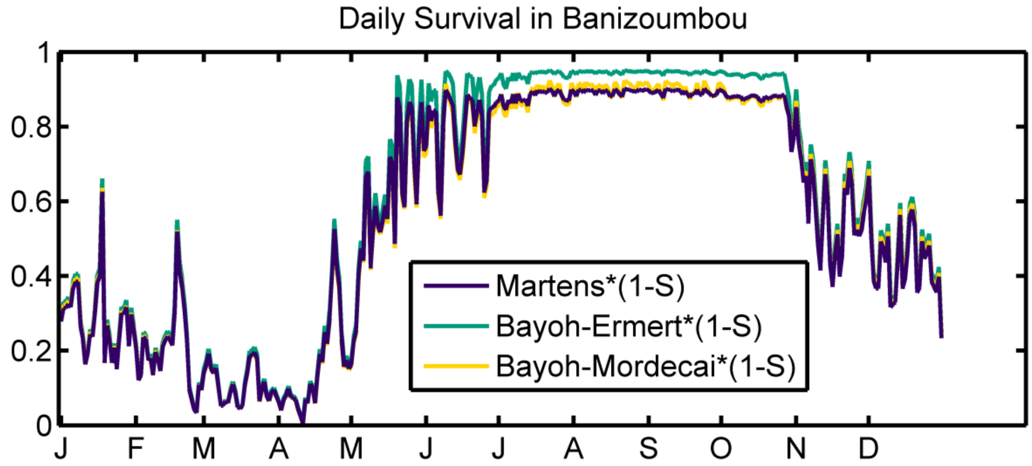

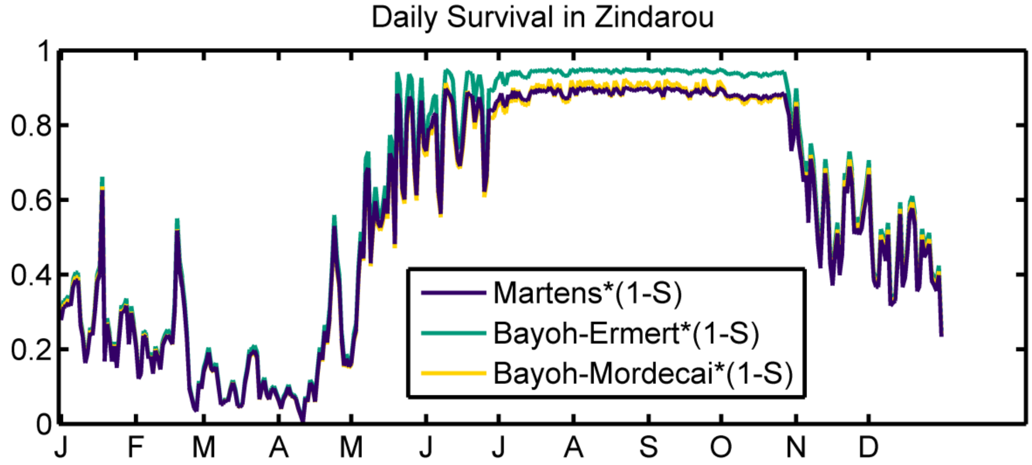

Figure S1.2: Daily probability of survival as a function of temperature and relative humidity, *p(T,RH)* using three different formulations for *p(T)*.

References

1. Martens WJ: **Health impacts of climate change and ozone depletion: An eco-epidemiological modelling approach.** *PhD thesis.* University of Maastricht, Dept. of Mathematics, Maastricht; 1997

2. Bayoh MN: **Studies on the development and survival of Anopheles gambiae sensu stricto at various temperatures and relative humidities.** *PhD thesis.* University of Durham, Durham; 2001

3. Ermert V, Fink A, Jones A, Morse A: **Development of a new version of the Liverpool Malaria Model. I. Refining the parameter settings and mathematical formulation of basic processes based on a literature review.** *Malar J* 2011, **10**(1):35.

4. Mordecai EA, Paaijmans KP, Johnson LR, Balzer C, Ben-Horin T, Moor E, McNally A, Pawar S, Ryan SJ, Smith TC: **Optimal temperature for malaria transmission is dramatically lower than previously predicted.** *Ecol Lett* 2013, **16**(1):22-30.
