# Supplementary figures and images for: Incorporating the effects of humidity in a mechanistic model of Anopheles gambiae mosquito population dynamics in the Sahel region of Africa
Source: Parasit Vectors. 2013 Aug 9;6:235. doi: 10.1186/1756-3305-6-235 (PMC3750695; doi:10.1186/1756-3305-6-235)

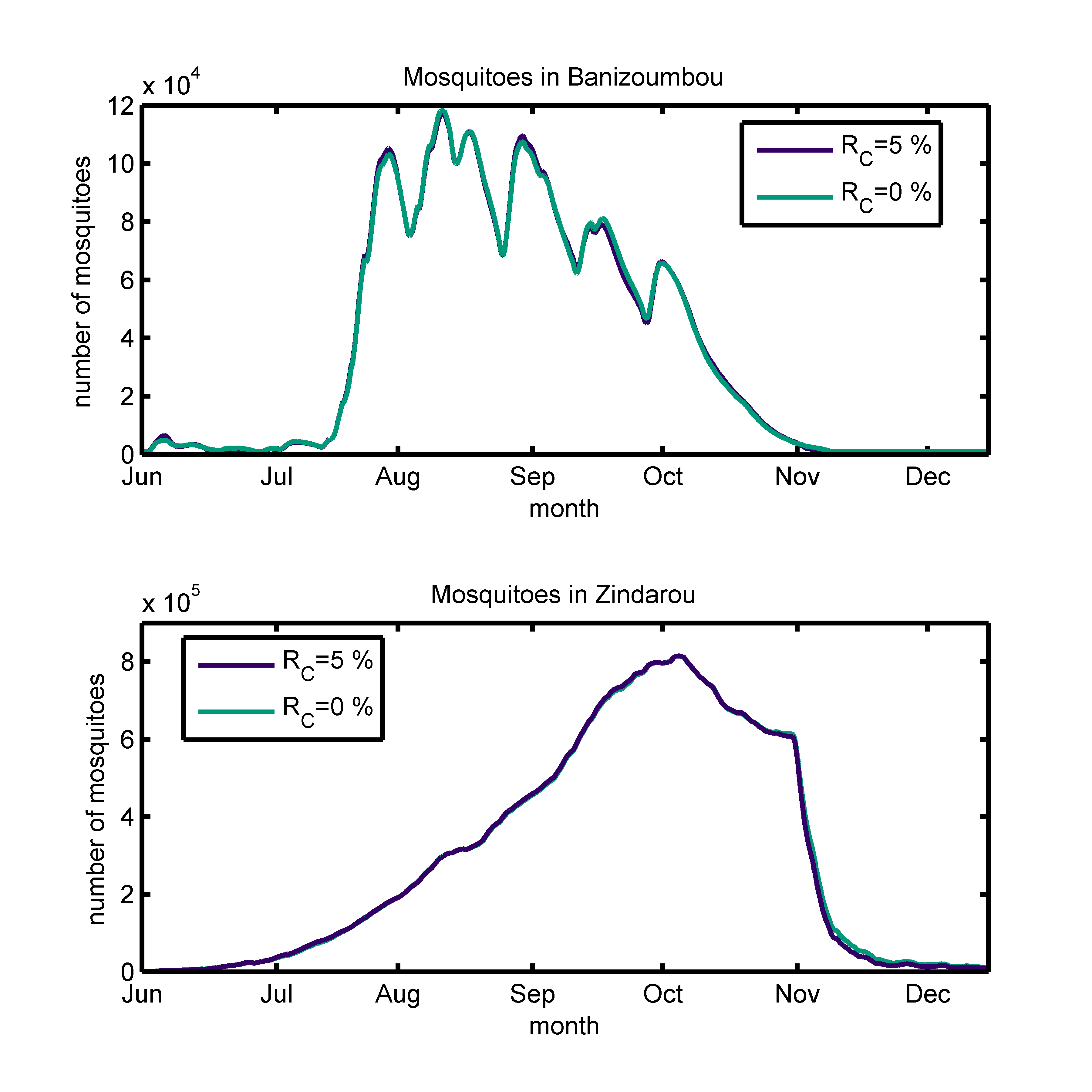

Supplement: Additional file 3 — Simulated mosquitoes in Banizoumbou and Zindarou with RHC= 5% and RHC = 0%. [file 1756-3305-6-235-S3.tiff]
